# The Effect of Foot Massage on Pain of the Intensive Care Patients: A Parallel Randomized Single-Blind Controlled Trial

**DOI:** 10.1155/2020/3450853

**Published:** 2020-06-13

**Authors:** Masoumeh Momeni, Mansoor Arab, Mahlagha Dehghan, Mehdi Ahmadinejad

**Affiliations:** ^1^Student Research Committee, School of Nursing and Midwifery, Kerman University of Medical Sciences, Kerman, Iran; ^2^Faculty of Nursing and Midwifery, Bam University of Medical Sciences, Bam, Iran; ^3^Nursing Research Center, Department of Critical Care Nursing, School of Nursing and Midwifery, Kerman University of Medical Sciences, Kerman, Iran; ^4^Department of Critical Care Medicine, Kerman University of Medical Sciences, Kerman, Iran

## Abstract

**Materials and Methods:**

This randomized, parallel, single-blind controlled trial study was performed on 75 ICU patients. Patients were allocated into three groups (massage by a nurse, massage by the patient's family, and control group) by the minimization method. Swedish massage was provided for the patients in experimental groups (each foot for 5 minutes) once a day for six days. The pain was examined in all three groups before, immediately, and one week after the intervention.

**Results:**

The mean scores of pain in the groups of foot massage by the patient's family and by a nurse showed a significant reduction at the end of the study (from 4.48 to 3.36 and 4.76 to 2.96, respectively). The control group had significantly more pain after the intervention than the family-based massage group and the nurse-based massage group (*P* < 0.05). Although significant difference was found in the mean scores of pain between the massage provided by a nurse and that provided by the patient's family immediately after the intervention (*P* < 0.05), it was not significant one week after the intervention (*P* > 0.05).

**Conclusion:**

Using foot massage, by both nurses and family members can reduce the pain of ICU patients. This intervention may improve the nursing care quality with the least cost and complications.

## 1. Introduction

Pain is an unpleasant sensory, emotional, and mental experience after real or potential tissue damage [1, 2]. Pain in the intensive care unit (ICU) is more common than that in the general wards, with 45%–82% of the ICU patients undergoing different degrees of pain [3]. The ICU patients' pain may not be treated due to low levels of consciousness, sedative drugs, and mechanical ventilation [1]. Pain and anxiety affects the nervous system resulting in the release of catecholamines and the formation of dysrhythmia, changes in vital signs, myocardial ischemia, mental disorders and delirium, delayed wound healing, poor sleep quality, agitation, patient's fighting the ventilator, and removal of catheters by the patient, a prolonged stay in ICU, re-admission, patient's dissatisfaction with medical care, and ultimate illness and increased mortality [4, 5]. During ICU treatment, 40–68% of patients experience moderate to severe pain [6, 7]. Puntillo et al. reported that half of the procedures in ICUS such as endotracheal and tracheal suctioning, chest tube removal, wound drain removal, turning, and arterial line insertion, increased the risk of patients having higher degrees of pain distress by at least 30% [8]. In addition, 38.2–66% of the intensive care survivors experience chronic pain up to one year after discharge [9, 10]. Therefore, the perception of a stressful experience is a key to recover from a critical illness: staff and family members should carefully evaluate different stressors while trying to avoid over- and underestimation [11].

Although pain activates a large number of pathophysiological mechanisms in the ICU patients, the rate of pain assessment in the ICU is low [12]. Rose et al. reported that nurses did not tend to use pain assessment tools in verbally disabled patients, and they had little information about new guidelines of pain controls that can have a negative effect on their performance in controlling the pain of patients [13]. Although recent guidelines support analgesics before nursing and medical procedures, it is unusual to administer analgesics before procedures [14, 15]. However, 30–60% of the ICU patients receive deep and long-term sedatives [16]. Opioids are the most frequently used drugs for pain relief in the ICUs. In addition, nonsteroidal anti-inflammatory drugs can be used to reduce the pain of the ICU patients [17]. Recently, light sedatives have been increasingly taken due to negative consequences of deep sedatives such as increased anxiety, stress, delirium, long stay, and infection risk [18, 19]. In addition, nonpharmaceutical methods can be used: aromatherapy, acupuncture, music therapy, biofeedback techniques, massage therapy, relaxation techniques, and reflexology [20].

In recent years, complementary medicines have been widely considered accompanying the standard treatment. Massage therapy, a type of complementary medicine, stimulates the nerves, tactile receptors and sends nervous impulses to the brain [21]. Massage reduces the patient's blood pressure and heart rate and makes him/her feel comfortable and relaxed [22]. Following the relaxation of the muscles, the endorphin is produced that improves sleep quality and alleviates pain and muscle cramps, and thus a pleasant sense is created and the need for sedatives reduces [23].

Different studies assessed the effect of massage on pain-relieving in the ICU patients. The results showed the effect of different types of massage therapies on pain relief of the ICU patients [20, 24–26]. Also, many studies have shown the role of family members in the care of ICU patients [27, 28]. A good health outcome in family-centered care approach will be achieved when the family members of the patient play an active role in physical, psychological, and emotional and social support [29]. Black et al. argued that the family-centered care strengthened the patient's defense lines, increased his/her resistance against stressors during acute illness, and improved his/her mental health [30]. Studies show that families receive more attention, support, and collaboration from nursing staff when participating in patient care [31–33].

Regarding the importance of evaluating and controlling pain in intensive care units and considering the destructive effects of sedatives and narcotics, complementary medicines can alleviate the pain of these patients [34]. Nurses can use nonpharmacological methods such as massage in intensive care units to control pain because they are simple, inexpensive, and easy-to-use. However, few studies have compared the effect of massage by nurses and family members on pain of the ICU patients. Regarding the nurses' workload and shortage, family involvement in the patient care may improve the patients' quality of care. Therefore, the present study aimed to compare the effect of foot massage by a nurse and the patient's family on pain of the ICU patients.

## 2. Materials and Methods

### 2.1. Study Design and Setting

This randomized, parallel, single-blind controlled trial has been conducted in the ICUs of Shahid Bahonar hospital in Kerman in 2017. Shahid Bahonar hospital, a trauma ICU center in southeastern Iran, has three trauma intensive care units and 46 beds.

### 2.2. Sample Size and Sampling

**Figure 1:**
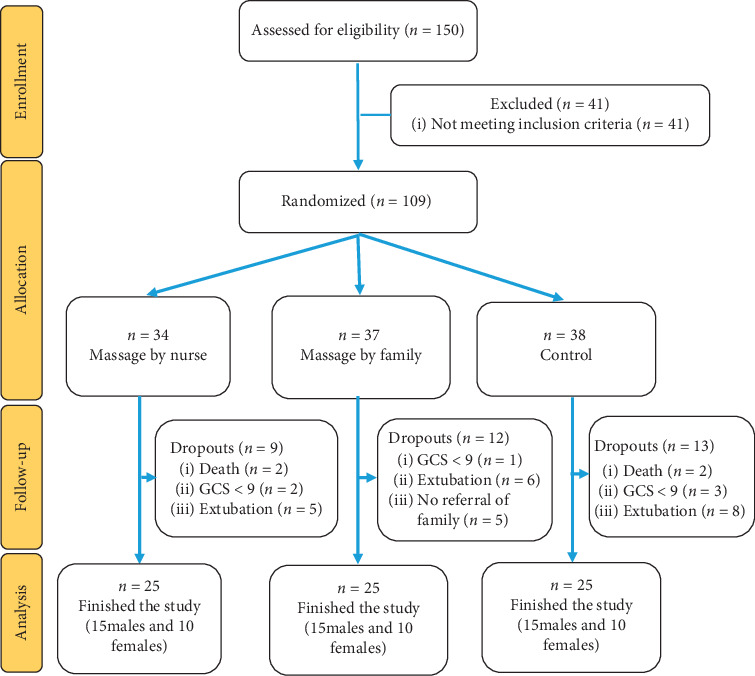
The flow diagram of the study.

The samples were selected using consecutive sampling method, and they were allocated into three groups (two groups of intervention and one control group) by stratified randomization method. The patients were allocated to each group after being checked in terms of sex and addiction history and then by drawing dice. This process continued until the end of sampling. The first author enrolled the participants and assigned them to the three groups. The inclusion criteria were as follows: patients who were supposed to be in the ICUs at least for two weeks, patients with tracheal tube or tracheostomy [35], discontinuous infusion of sedative drugs such as midazolam and propofol [36], discontinuous infusion of narcotics such as fentanyl [27], patients aged between 15 and 50 years [35], with no withdrawal syndrome [37], no foot vascular disease, such as deep venous thrombosis [38], no fractures in the lower extremities [38], no skin disease, ulcers, and infections of feet [19]. Exclusion criteria include the patient's extubation [35], reduction in the level of consciousness to less than 9 due to hypoxia or/and increased intracranial pressure [37], no referral of the patient's family to the ward for massage for more than two days, patient's death, and discharge [35]. Previous studies were used for the estimation of sample size [39]. The confidence coefficient, the confidence interval, and the type-II-error were 95%, 1.96, and 10%, respectively (the study power = 90%). According to the three study groups, the sample size was adjusted and the number of samples needed for this study was 21 in each group. However, 25 samples were considered in each group (Figure 1) to improve the study power.

### 2.3. Measurements

The data-gathering tool in this study was the sociodemographic and clinical information questionnaire and behavioral pain scale (BPS). The sociodemographic and clinical information questionnaire included age, sex, marital status, occupation, level of education, history of admission to intensive care units, history of other illnesses, history of addiction, history of smoking, history of seizure, history of hypertension, duration of stay in the ICU, history of surgery during hospitalization, type of disease, and vital signs. BPS was used to assess pain. Payen et al. developed BPS in 2001. The scale has three items: facial appearance, status of upper limbs, and ventilator compatibility. Dehghani et al. investigated the validity and reliability of the scale. These results showed that the scores of behavioral pain scale were significantly different during painful and nonpainful procedures. Also, Cronbach's alpha was 85 for painful procedures and 76 for nonpainful procedures [40]. Yaghoubiana et al. reported the reliability of this scale to be 0.86 [20].

### 2.4. Data Collection and Intervention

The researcher went to Shahid Bahonar hospital in Kerman and obtained informed consent from the patient's family after receiving the code of ethics and the clinical trial registration code as well as coordinating with the head of the hospital and the authorities of the intensive care units. The coresearcher received sufficient training and measured the pain variable in three groups before the massage without being aware of the samples assignment. A physiotherapist trained the first researcher (ICU nurse) how to massage. In the intervention groups, in addition to routine care, a nurse or a family member of the patients performed Swedish massage on their feet (from knee to toes) for six consecutive days for 10 minutes (5 minutes for each foot). The massage procedure was as follows: first, the patient was placed in a supine position with a pillow under the feet so that the feet were bent slightly and the head was at an angle of 30–45 degrees. The massage area was uncovered from 10 cm above the patient's knee. Then, the researcher (the nurse) began to massage after examining the feet for the presence of the massage barriers. The Swedish massage included stroking, effleurage, vibrations, or kneading. Baby oil was used to make the area slippery and easy-to-massage (about 1-2 cc per foot), and it had no other therapeutic value. The massage was performed at 15–17o'clock when the workload of the intensive care unit was low. A napkin was used to remove the residual oil on the patient's body. The first researcher, who was a nurse of the intensive care unit, performed all massages. In addition, she trained one or two family members of the patient how to massage, and they initiated the intervention after her confirmation. The control group only received routine care. The pain was reexamined in all three groups immediately (sixth day) and one week after the intervention (thirteenth day). A coresearcher, who was not aware of the patients' assignment, assessed all measurements.

### 2.5. Data Analysis

Data were analyzed by SPSS 18. Descriptive statistics (frequency, percentage, mean, and standard deviation) were used to describe patients' demographic characteristics and clinical information. Mean and standard deviation were used to describe the pain score. Chi-squared test, Fisher's exact test, Kruskal–Wallis test, and ANOVA were used to examine the similarity of the three groups regarding the study variables. Regarding the establishment of parametric conditions (normal distribution and equality of variances in the three groups), the two-way factorial ANOVA test was used to compare the pain score before, immediately, and one week after the intervention within and between the three groups. The post hoc test of Bonferroni used for multiple comparisons.

### 2.6. Ethical Considerations

Kerman University of Medical Sciences approved the study protocol (No. ir.kmu.rec.1396.1460). The study protocol was registered to the Iran RCT center (No. IRCT201707317844N12). The researcher obtained informed consent from the patient's guardian.

## 3. Results

The mean age of the samples in the family-based massage group was 41.12 ± 9.29, and it was 39.49 ± 10.79 in the nurse-based massage group and 42.8 ± 6.65 in the control group. No statistically significant difference was found among the three groups in the mean age (*P*=0.42). Sixty percent (*n* = 15) of the samples were men. Sixty-eight percent (*n* = 17) of the samples in the family-based massage group, 76% (*n* = 19) of the samples in the nurse-based massage group, and 84% (*n* = 21) of the samples in the control group were married (*P*=0.42: *χ*^2^ = 1.75). In addition, no statistically significant difference was found among the three groups in occupation (*P*=0.96) and education levels (*P*=0.24). No differences were observed in the medical history among the three groups (Table 1).

The level of consciousness was nine in all three groups based on the Glasgow coma scale. The length of stay in the ICU was five to six days in all three groups before the study (*P*=0.06). Eighty-four percent of the samples in the family-based massage group, 88% in the nurse-based massage group, 92% in the control group had the tracheal tube, and the rest had a tracheostomy (*P*=0.90). No statistically significant difference was found among the three groups in the rate of spontaneous breathing (*P*=0.49), the rate of assisted spontaneous breathing (*P*=0.59), the rate of inspired oxygen (*P*=0.37), and the positive end-expiratory pressure (*P*=0.90). The administration of narcotics (midazolam, propofol, morphine, methadone, fentanyl, and opium) and nonnarcotics (nitroglycerin, metoral, hydrochlorothiazide, propranolol, and dopamine) was not significantly different among the three groups before the intervention (*P* > 0.05). In addition, no significant differences were found among the three groups in the mean arterial blood pressure (*P*=0.28), heart rate (*P*=0.08), and arterial blood saturation (*P*=0.61) at the beginning of the study.

The mean score of pain in the three groups at different times is presented in Table 2. The results of two-way ANOVA showed a statistically significant group-time interaction which indicates that the pain scores were different between groups at different times (Table 3). The within-subject comparisons showed, in the control group, the pain alleviation at the different times were not significant. In both massage groups, the pain alleviation immediately and one week after intervention compared with that before intervention was significant; however, the pain alleviation immediately after intervention compared with that one week after intervention was not significant (Table 4).

Multiple comparisons showed the pain scores were not different between groups before the intervention. Immediately after intervention, patients in the nurse-based massage group had significantly less pain than those of the family-based massage group and the control group. One week after intervention, patients in the nurse-based massage group and the family-based massage group had significantly less pain than those of the control group. One week after intervention, there were no differences between the two massage groups (Table 5).

## 4. Discussion

The results of the present study showed that the mean score of pain in patients admitted to intensive care units was mild. A six-day foot massage either by the family or a nurse significantly reduced the mean score of pain compared with routine care (control group). Few studies compared the effect of massage performed by the family and a nurse on pain of the ICU patients [25]. Jamaati et al. showed that the whole-body massage by a nurse and the patient's family for 30 minutes reduced the pain score in the ICU patients with the consciousness level of 10–15 [25]. Similar to the results of this study, massage reduced pain in the study of Jamaati et al. However, unlike the results of this study, family-based massage had a greater effect on the pain-relief compared with the nurse-based massage [29]. The reason for the differences might be the LOC of ICU patients.

Several studies have examined the effect of massage by the family or a nurse on pain of the ICU patients. Najafi et al. showed a reduced pain intensity in patients undergoing coronary artery bypass graft surgery after being massaged by the their family caregivers [41]. Cutshall et al. showed that the Swedish massage of the whole body after the coronary artery bypass graft surgery significantly reduced the pain, anxiety, and tension of the muscles [42]. Yaghoubinia et al. showed that the foot massage reduced the pain score in unconscious ICU patients [20]. Braun et al. found that the Swedish massage decreased the pain after the surgery compared with that of the control group [26]. Babajani et al. studied the pain intensity while removing a chest tube after open-heart surgery and showed that the pain score reduced in foot reflexology massage groups compared with the control and placebo groups [24]. Also, Imani et al. showed that reflexology massage could reduce the intensity of nitroglycerin-induced migraine-type headache in the CCU patients [43]. The results of the above studies support the results of the present study. Although there are many differences among the patients in various studies, the type, duration and areas of the massage, and pain measuring instruments, all studies confirmed the effect of massage on pain. Several theories have been proposed to describe the mechanisms of massage therapy. One is the gate control theory for pain relief suggesting that massage has an analgesic effect [44]. The second one is that massage can relax the patient and reduce pain by improving the secretion of endorphins, increasing the activity of the parasympathetic system, and decreasing the production of stress hormones [23, 45]. The third theory suggests that manipulating the skeletal-muscular system releases tension from muscle fibers and connective tissue [46].

The results of the present study and available evidence suggest that massage by trained nurses or family members of patient can reduce the pain of ICU patients. Therefore, considering the importance of family-centered care, nurses' workload, and shortage, families can provide massage, and involving families in massages can lead to effective interventions after patients' discharge from the hospital. However, nurses should train patients and their families properly [32, 41]. In addition, training different skills of complementary and alternative methods, particularly massage to nursing students may improve their ability to provide better nursing care for the patients.

One of the limitations of this study was the lack of cooperation of some families. No information about the disease and the effect of massage on the patient and difficulty to attend in the hospital for six consecutive days were the causes of this limitation. This limitation was eliminated by training the family and reducing their concerns as well as by contacting with families who were not present at a proper time. In addition, two family members who provided the massages might influence the results. However, only seven cases were massaged by two family members (3-4 sessions with one family member and 1-2 sessions with another member). As the majority of the sessions were provided with the same family members, the data were analyzed on a per-protocol basis and not an intention to treat. The study samples did not represent all patients in the intensive care unit, so the results could not be generalized to other patients admitted to the intensive care unit. In addition, the first researcher allocated the samples into groups, which may be associated with bias.

## 5. Conclusion

According to the results of this study, our hypothesis is approved. On the other hand, the mean scores of pain was significantly different among the family-based massage group, the nurse-base massage group, and the control group after the intervention. The mean pain scores in the family-based massage group and the nurse-based massage group significantly decreased after the intervention, but the mean pain score in the control group did not significantly decrease after the intervention. Massage, an easy and low-cost method, is helpful for pain reduction in unconscious patients admitted to the intensive care units. Regarding the importance of pain control in patients admitted to the intensive care units and considering few studies in the field of complementary medicine and massage therapy on patients admitted to ICU, researchers interested in the field of complementary medicine are recommended to do studies with a larger sample size in different environments and research communities. Further studies are recommended to clarify the effect of massage therapy on other symptoms of unconscious patients admitted to the intensive care units, including agitation, delirium, and intensive care unit-acquired weakness.

## Figures and Tables

**Table 1:** Comparison of the medical history in the family-based massage group, nurse-based massage group, and control.

Variable	Group							
	Family-based massage group		Nurse-based massage group		Control group		Statistical test	*P* value
	Frequency	Percent	Frequency	Percent	Frequency	Percent		
History of admission in ICU								
Yes	2	8.0	4	16.0	2	8.0	1.08^*∗*^	0.72
No	23	92.0	21	84.0	23	92.0		

History of other diseases								
Yes	11	44.0	12	48.0	12	48.0	0.11^*∗∗*^	>0.99
No	14	56.0	13	52.0	13	52.0		

Current history of addiction								
Yes	11	44.0	11	44.0	11	44.0	—	—
No	14	56.0	14	56.0	14	56.0		

History of smoking								
Yes	9	36.0	7	28.0	9	36.0	0.48^*∗∗*^	0.86
No	16	64.0	18	72.0	16	64.0		

History of seizure								
Yes	0	0.0	3	12.0	0	0.0	4.26^*∗*^	0.10
No	25	100.0	22	88.0	25	100.0		

Hypertension								
Yes	10	40.0	10	40.0	9	36.0	0.11^*∗*^	0.94
No	15	60.0	15	60.0	16	64.0		

History of taking hypertension medications								
Yes	10	40.0	9	36.0	9	36.0	0.11^*∗∗*^	0.99
No	15	60.0	16	64.0	16	64.0		

History of surgery during hospitalization								
Yes	21	84.0	20	80.0	20	80.0	0.26^*∗*^	0.99
No	4	16.0	5	20.0	5	20.0		

Type of disease								
Lesions with bleeding	6	24.0	9	36.0	9	36.0	1.10^*∗∗*^	0.70
Lesions without bleeding	19	0.76	16	0.64	16	0.64		

^*∗*^Fisher's exact test; ^*∗∗*^Chi-squared test.

**Table 2:** The pain score in the family-based massage group, nurse-based massage group, and control at different times.

Pain score	Group		
	Family-based massage group	Nurse-based massage group	Control group
	Mean (SD)	Mean (SD)	Mean (SD)
Before intervention	4.48 (0.77)	4.76 (1.13)	4.28 (1.14)
Immediately after intervention	3.64 (0.70)	2.88 (0.78)	4.20 (0.76)
One week after the intervention	3.36 (0.64)	2.96 (0.67)	4.00 (0.71)

SD: standard deviation.

**Table 3:** Comparison of pain in the family-based massage group, nurse-based massage group, and control at different times.

Source of change	Sum of squares	d*f*	*F*	*P* value	Partial Eta squared
Group	14.75	2	10.69	<0.001	0.09
Time	50.67	2	36.74	<0.001	0.25
Group*∗*Time	23.87	4	8.65	<0.001	0.14
Error	148.96	216			

**Table 4:** Adjustment for multiple comparisons using Bonferroni for the comparison of pain within the family-based massage group, nurse-based massage group, and control at different times.

Group	Time (*I*)	Time (*J*)	Mean difference (*I* − *J*)	Standard error	*P* value	95% confidence interval for difference
Family-based massage group	Before	Immediately after intervention	0.84	0.24	0.001	0.27–1.41
		One week after the intervention	1.12	0.24	<0.001	0.55–1.69
	Immediately after intervention	One week after the intervention	0.28	0.24	0.70	−0.29–0.85

Nurse-based massage group	Before	Immediately after intervention	1.88	0.24	<0.001	1.31–2.45
		One week after the intervention	1.80	0.24	<0.001	1.23–2.37
	Immediately after intervention	One week after the intervention	−0.08	0.24	>0.99	−0.65–0.49

Control group	Before	Immediately after intervention	0.08	0.24	>0.99	−0.49–0.65
		One week after the intervention	0.28	0.24	0.70	−0.29–0.85
	Immediately after intervention	One week after the intervention	0.20	0.24	>0.99	−0.37–0.77

**Table 5:** Adjustment for multiple comparisons using Bonferroni for the comparison of pain between the family-based massage group, nurse-based massage group, and control at different times.

Time	Group (*I*)	Group (*J*)	Mean difference (*I* − *J*)	Standard error	*P* value	95% confidence interval for difference
Before	Family-based massage group	Nurse-based massage group	0.28	0.24	0.70	−0.85–0.29
		Control group	0.20	0.24	>0.99	−0.37–0.77
	Nurse-based massage group	Control group	0.48	0.24	0.13	−0.09–1.05

Immediately after intervention	Family-based massage group	Nurse-based massage group	0.76	0.24	0.004	0.19–1.38
		Control group	−0.56	0.24	0.05	−1.13–0.007
	Nurse-based massage group	Control group	−1.32	0.24	<0.001	−1.89 to −0.75

One week after the intervention	Family-based massage group	Nurse-based massage group	0.40	0.24	0.27	−0.17–0.97
		Control group	−0.64	0.24	0.02	−1.21 to −0.07
	Nurse-based massage group	Control group	−1.04	0.24	<0.001	−1.61 to −0.47

## Data Availability

The data used to support the findings of this study are available from the corresponding author upon request.
